# Fused deep learning enables 6D single-molecule localization in polarization-resolved microscopy

**DOI:** 10.1088/2050-6120/ae8eff

**Published:** 2026-08-10

**Authors:** Emil Gillett, Subhojyoti Chatterjee, Jagriti Chatterjee, Nikita Kovalenko, Cong Xu, Dongyu Fan, Yiyang Chen, Yuanxin Qiu, Junyuan Miao, Varun Nelavoy, Matthew D Lew, Mikael P Backlund, Christy F Landes

**Affiliations:** 1 Department of Chemistry, University of Illinois Urbana-Champaign, Urbana, IL 61801, United States of America; 2 Department of Electrical and Systems Engineering, Washington University in St. Louis, St. Louis, MO 63130, United States of America; 3 Department of Chemical and Biomolecular Engineering, University of Illinois Urbana-Champaign, Urbana, IL 61801, United States of America; 4 Department of Electrical and Computer Engineering, University of Illinois Urbana-Champaign, Urbana, IL 61801, United States of America

**Keywords:** optical microscopy, orientation, deep learning, super-resolution, fluorescence, double-helix, phase engineering

## Abstract

Single-molecule orientation localization microscopy (SMOLM) is an optical means to measure complex transport in charged and crowded conditions, such as inside cells or polymer materials. SMOLM extracts time- and space-dependent three-dimensional orientation information from dipole emitters. Achieving simultaneous position-orientation resolution with high photon efficiency remains a central challenge in SMOLM instrument design. We developed an optical fluorescence microscope that uses the double-helix point spread function (DHPSF) to localize dipole emitters in six dimensions (6D), delineated by spatial and dynamic orientational parameters. Furthermore, we developed a fused deep learning approach based on existing neural network architectures to localize dipole emitters in 6D. Our microscope enables simultaneous 6D localization of single fluorophores, achieving a median spatial precision of 10 nm and angular precision below 10° across most of orientation space, except for the azimuthal angle at high polar angles where the DHPSF exhibits known optical degeneracies. We demonstrate our approach by localizing single rhodamine B molecules in poly(methyl methacrylate) films. The recovered orientations show $\theta $ near 90° and small wobble angles. We also demonstrate 6D SMOLM of a spherical supported lipid bilayer, where despite the low signal, out-of-training distribution of the experimental data, we observe clearly ordered orientation of Nile red molecules within the membrane.

## 1. Introduction

Single-molecule localization microscopy (SMLM) [1–3] has advanced the ability to resolve fluorescent emitters beyond the diffraction limit, down to nanometer length scales. SMLM utilizes the photophysical properties of fluorescent molecules to probe nanoscale environments, yielding insights into cellular processes [4, 5], disease mechanisms [6–9], and material properties [10–21]. The extension of SMLM to single-molecule orientation localization microscopy (SMOLM) provides the ability to understand the distinct contributions of orientational and rotational mobility, especially in biological systems [22, 23].

A combination of existing methods such as emission pattern imaging [24, 25], polarized detection [5, 26–28], and reflector-based geometries [29] has advanced the ability to measure dipole orientation [30]. Calcite-Assisted Localization and Kinetics microscopy localizes fluorophores in the lateral ($x$, $y$) plane with precision down to ≈10 nm and recovers the in-plane orientation of dipole emitters [31]. POLCAM [32] simplifies SMOLM using a polarization camera, eliminating the need for additional polarization optics that can recover the 3D spatial coordinates ($x$, $y$, $z$) and the 3D orientations ($\theta $, $\phi $, ${{\Omega }}$) of dipole emitters. However, these approaches that extract 6D information typically require additional steps, such as axial scanning of the objective to obtain the axial coordinate or require extra optical elements that complicate system alignment.

Phase engineering modulates the shape of diffraction-limited emission patterns, or point spread functions (PSFs), enabling the encoding of axial depth [33–36], spectral information [37–39], sub-frame temporal information [40, 41], or orientation [30, 42–46] with increased sensitivity. To address the challenges posed by the prevalence of shot noise [47] and photon budget limitations [48] in SMLM and SMOLM, artificial intelligence has been widely adopted for estimating position and orientation from phase-engineered PSFs in low signal-to-noise conditions. Machine learning classifiers applied to single‐particle trajectories can reliably distinguish receptor-ligand interaction states and diffusion regimes [49] and classify 3D single molecule trajectories into different motion types [50]. Deep learning (DL) frameworks [30, 51–57] have improved accuracy and throughput for analyzing the 3D positions and orientations of overlapping, phase-engineered PSFs. One such phase-engineered design, the double-helix PSF (DHPSF), is a robust 3D PSF model that encodes axial position as the rotation angle between two bright lobes, enabling precise 3D single-molecule localization [58]. Its compact footprint minimizes errors from overlapping emitters, making the DHPSF suitable for imaging complex 3D environments such as cells [33] and polymer films [42]. These efforts highlight the value of approaches that combine artificial intelligence with fluorescence microscopy and phase‐engineering with a goal to simultaneously preserve high precision and address the technical trade‐offs inherent in SMOLM.

In this work, we present an integrated optical system that combines advanced phase modulation and polarization-resolved imaging with fused DL. Our approach enables high-precision 6D localization: 3D position, and 3D orientation using a double-helix (DH) phase mask and a polarizing beam splitter (PBS) [42]. We fused DL outputs by combining the 5D capability of Deep-SMOLM ($x$, $y$, $\theta $, $\phi $, ${{\Omega }}$) [30], and the 3D capability of DeepSTORM3D ($x$, $y$, $z$) [52] to extract 6D coordinates. We demonstrate our approach by localizing single rhodamine B (RhB) fluorescent dye molecules embedded within poly(methyl methacrylate) (PMMA) films. We validated our analysis and confirmed the accuracy of emitter recognition (Jaccard index, a measure of detection fidelity [59, 60], of 0.74) within polymer matrices, highlighting the effectiveness of our methodology for SMOLM. This fused optical-computational framework achieves 6D localization with high precision across diverse emitter orientations and positions. This method provides a scalable and experimentally accessible platform for studying structural dynamics at a single-analyte level in biological, separations, and energy storage systems.

## 2. Materials and methods

### 2.1. Microscope coverslip preparation

Microscope coverslips (#1, 22$ \times $ 22 mm, Fisher) were cleaned by sequentially sonicating them in a detergent solution (Liquinox, 30 min), deionized water (30 min), methanol (15 min), and acetone (15 min). After these consecutive cleaning steps, the glass coverslips were placed in a heated, alkaline piranha solution (1:1:5 ratio of 30% NH_4_OH:30% H_2_O_2_:H_2_O at 80 °C). The coverslips were then dried under a stream of compressed nitrogen and stored in an evacuated desiccator. Immediately before each experiment, the coverslips were removed from the desiccator and plasma-cleaned with oxygen for 2 min (Harrick Plasma, PC-32 G).

### 2.2. Supported lipid bilayer-coated silica beads

Large unilamellar vesicles were prepared from a mixture of 1,2-dipalmitoyl-sn-glycero-3-phosphocholine (DPPC, Avanti Polar Lipids) and cholesterol (Avanti Polar Lipids) at concentrations of 0.29 mg ml^−1^ DPPC and 0.1 mg ml^−1^ cholesterol (40 mol% cholesterol) in Tris-Ca^2+^ buffer (10 mM Tris, 100 mM NaCl, 3 mM CaCl_2_, pH 7.4) by the extrusion method using a Mini-Extruder (Avanti Polar Lipids) fitted with a 100 nm polycarbonate membrane (Whatman, Nuclepore) at 65 °C. To coat 2 *µ*m silica beads (Bangs Laboratories, SS04002) with supported lipid bilayers, 1 *µ*l of bead stock (2 g ml^−1^) was mixed with 149 *µ*l of Tris-Ca^2+^ buffer, vortexed for 20 s, and centrifuged at 2000 rpm for 5 min. The top 100 *µ*l of supernatant was removed, and the bead pellet was diluted in Tris-Ca^2+^ buffer to a final concentration of 2.78 mg ml^−1^. The washed beads (90 *µ*l) were then combined with the DPPC + 40 mol% cholesterol vesicle solution (90 *µ*l, pre-warmed to 65 °C), incubated in a 65 °C water bath for 30 min with periodic vortexing every 5 min, and slowly cooled to room temperature over 1.5 h with continued periodic vortexing to ensure uniform bilayer formation. The bilayer-coated beads were washed six times by centrifugation at 600 rpm for 5 min, with each cycle replacing 120 *µ*l of supernatant with 120 *µ*l of Ca^2+^-free Tris buffer (10 mm Tris, 100 mm NaCl, pH 7.4) to remove unbound vesicles and Ca^2+^ ions. For imaging, beads were diluted 2 880-fold in water and stained with 0.5 nM Nile red (Sigma-Aldrich) in a Press-To-Seal silicone isolator chamber (Grace Bio-Labs JTR24R-A-1.0; 24 wells, 3 mm diameter $ \times $ 0.9 mm depth) adhered to a clean coverslip.

### 2.3. PMMA film preparation

Powdered PMMA (MW = 15 kD) and high-performance liquid chromatography-grade toluene were purchased from Sigma-Aldrich. PMMA was dissolved in toluene at 20 wt%, sealed in a flask, and stirred overnight, with a portion set aside without RhB for the first film layer. A 1 M stock of RhB fluorescent dyes (Sigma) was diluted to 8.3 nM in toluene, then to 165 pM in the PMMA-toluene solution. 100 *μ*l of the PMMA solution without RhB was pipetted onto the prepared coverslip surface and spin-coated at 2.0$ \times $ 10^3^ rpm for 30 s, then at 6.0$ \times $ 10^3^ rpm for another 30 s (WS-650MZ-23NPPB, Laurell). The coverslips were then positioned in the path of the 532 nm laser at 80 mW for 24 h to photobleach the imaging area. A representative movie illustrating the film after this 24 h photobleaching step is provided in supplementary movie 1. After photobleaching, the second layer of 20 wt% PMMA containing 165 pM RhB was spin-cast onto the first film layer using the same spin-coating settings described above.

### 2.4. Ellipsometry

The film thickness and refractive indices were obtained via ellipsometry (RC2, J. A. Woollam). PMMA films were prepared on glass coverslips as described above. Film thickness and refractive index measurements were made over a 25-point grid across the coverslip surface. The ellipsometry data was fit to a bilayer Cauchy model for transparent dielectric films with intermix between Cauchy layers. The total thickness, ${T_{{\mathrm{tot}}}}$, of the film was calculated as the sum of the two layers, ${T_{L{\mathrm{1}}}}$ and ${T_{L2}}.$ The macroscale effective refractive index, ${n_{{\mathrm{eff}}}}$, was approximated with a thickness-weighted average (equation (1)):

\begin{equation*}{n_{{\mathrm{eff}}}} = \frac{{{n_{L1}}\left( \lambda \right){T_{L1}} + {n_{L2}}\left( \lambda \right){T_{L2}}}}{{{T_{L1}} + {T_{L2}}}}\end{equation*}

since both layers of PMMA were spun from toluene, the approximation collapses to the bulk PMMA index.

### 2.5. Microscope calibration

The PSF model was calibrated using a $z$ -stack of images of fluorescent beads fixed on a coverslip [61]. To generate training data and process data acquired by the microscope from two orthogonally polarized channels, calibration samples were prepared from a 1:1000 dilution of 0.1 *μ*m fluorescent polystyrene beads (carboxylate-modified, orange (540/560) FluoSpheres, Fisher), drop-cast on a cleaned coverslip, and dried under a stream of compressed nitrogen. The sample was mounted onto a piezoelectric stage (P-517.3CL stage, E-727 controller, Physik Instrumente) which was programmed to move from 2 *μ*m to −2 *μ*m in the $z$ coordinate while recording a movie. During the stage movement, the scientific complementary metal-oxide-semiconductor (sCMOS) detector (Prime95b, Teledyne Photometrics) recorded a movie. The recorded movie was pre-processed via custom-built image registration software, where the two channels were identified and colocalized (figure S1). After the colocalization step, the experimental phase masks encoded with aberrations were retrieved from each channel (figure S2) [62].

### 2.6. Instrument and acquisition parameters

The effective pixel size at the sample plane was 66 nm. At $z$ = 0 *μ*m, the PSF footprint was approximately 30 pixels in diameter. Excitation was provided by a 532 nm continuous-wave diode laser operated in continuous-wave mode (not camera-triggered). The measured laser power at the sample was 10.6 mW for experimental PMMA film measurements and 2.7 mW for calibration *z*-stacks. The beam diameter at the sample was 96 *μ*m (e^−2^), corresponding to a power density of 0.14 kW cm^−2^ during imaging. The half-wave plate used to rotate the vertical emission polarization was an achromatic half-wave plate (AHWP10M-600, Thorlabs). The reflective SLM (PLUTO VIS-130, Holoeye) had a reflectivity of 94% with a 93% fill factor. The back-illuminated sCMOS detector (Prime95b, Teledyne Photometrics) was operated in 16-bit mode with full-well sensitivity and rolling shutter readout. The camera integration time was 100 ms per frame. The average background photon count of 33 photons per pixel was estimated from the four 20$ \times $ 20-pixel corner regions of each 60$ \times $ 60-pixel crop across both channels, where no emitter signal was present, following the local background estimation approach used in vectorial implementation of phase retrieval. The full field of view spanned approximately 690$ \times $ 690 pixels, from which 60$ \times $ 60-pixel regions of interest (ROIs) were manually selected for analysis. A total of 75 movies of 200 frames each were acquired. A single frame index (frame 44) was randomly selected and applied uniformly across all movies to ensure consistent photobleaching conditions and reduced emitter density suitable for sparse single-molecule localization. For PSF calibration, the piezoelectric stage was stepped axially in 10 nm increments at a speed of 0.33 *μ*m s^−1^. During calibration, the sCMOS detector operated at a 30 ms integration time in post-sequence mode with an excitation laser power of 0.037 kW cm^−2^. For the DPPC-coated bead measurements, the excitation power density was 0.5 kW cm^−2^, the integration time was 100 ms, and a bandpass filter (Semrock AF01–585/29–25) was placed in the detection path.

### 2.7. Image preprocessing and localization workflow

For simulated data, images were generated with dimensions of 60$ \times $ 60 pixels with 1–3 randomly positioned emitters. For experimental data, 60$ \times $ 60-pixel ROIs were manually selected using a custom MATLAB graphical user interface. ROIs were chosen to contain visibly isolated PSFs with 1–3 emitters per crop, prioritizing regions where emitters were well-separated in both colocalized polarization channels to minimize crowding artifacts and ensure reliable single-molecule analysis. The 60$ \times $ 60-pixel dimension was selected to encompass the full PSF extent (including phase mask features) while maintaining computational tractability. Each ROI was processed through both neural networks independently producing 6D estimates that were fused via the algorithm described below. Manual ROI selection may bias the analysis toward well-isolated, higher-signal emitters. To address this limitation, an automated pipeline is under development that systematically partitions the full field of view from both polarization channels into an ROI grid with metadata preserving each region’s spatial origin, thus eliminating human selection bias.

### 2.8. Optical simulations for training and validation datasets

A synthetic dataset was generated using the forward model of the microscope with an upsampling ratio of 6 and a pixel size of 11 *μ*m. An expanded description can be found in the results and discussion section, with parameters specified in table S1. The dataset of 3.3$ \times $ 10^4^ images was generated with signals chosen randomly from a compound Poisson-Gaussian distribution with a mean of 5.5$ \times $ 10^3^ signal photons and an average background photon count of 33 photons per pixel. The simulated images were generated with random positions and orientations, with a minimum of 1 and a maximum of 3 emitters per image. The simulated images were generated with dimensions of 60$ \times $ 60 pixels, then allocated in a (75:15:10) ratio for training, validation, and testing, respectively.

### 2.9. Modified deepSTORM3D convolutional neural network (CNN) model architecture

A modified DeepSTORM3D (mDeepSTORM3D) framework was inspired by the original DeepSTORM3D [52] architecture, retaining its core design principles while it introduced targeted changes to handle multi-channel inputs and enhance feature integration. The principal modification introduced in mDeepSTORM3D relative to the original DeepSTORM3D architecture is the dual-channel input processing to accommodate polarization-resolved PSFs instead of a single scalar intensity channel. Further targeted architectural updates such as dilated convolutions (dilation rates = 2, 4, 8, 16), enhanced skip-connections, two-stage bilinear upsampling in the ($x$, $y$) plane ($ \times $ 4 total), and a residual convolutional head with HardTanh [63, 64] activation improved convergence stability and localization robustness, while maintaining the core principles of DeepSTORM3D.

The network was built from repeated convolutional processing units, each comprising a 2D convolution layer, a leaky rectified linear unit nonlinearity [65], and a batch normalization (BN) layer [66]. The convolution layers (with 3$ \times $ 3 kernels) acted as localized spatial filters that progressively extracted fine-grained spatial features from raw photon detection maps. The LeakyReLU activation mitigated neuron inactivity and preserved gradient flow even for weak photon signals, which was essential in sparse-emitter imaging. BN both stabilized training and mitigated internal covariate shift, which enabled faster convergence. When combined with skip-connections formed by concatenating the original input with intermediate feature maps, these Conv–LeakyReLU–BN units allowed the network to merge low-level spatial detail with increasingly abstract, multi-scale features. In deeper layers, dilated convolutions [67], which insert gaps between kernel elements to expand the receptive field without reducing resolution, enabled simultaneous capture of fine localization cues and long-range spatial context.

The localization CNN, implemented in PyTorch [68], was trained for 100 epochs, with early stopping triggered if no improvement in the validation loss was observed for 10 consecutive epochs. Optimization was performed using the Adam optimizer [69], and the kernel density estimation-based 3D localization loss function (equation S3) was employed to enhance spatial accuracy, with the Jaccard index tracked as a complementary metric for spatial overlap. A Jaccard index $ \ge $0.7 was considered indicative of high overlap consistency. A ReduceLROnPlateau [68] scheduler dynamically adjusted the learning rate based on the validation loss trend to prevent overfitting. During training, the model’s weights were periodically saved as checkpoints, with the best-performing models determined by minimum validation loss, maximum Jaccard index, and smallest training-validation loss gap. Real-time training and validation metrics were continuously logged and visualized, facilitating the monitoring of network convergence. The trained mDeepSTORM3D model was ultimately used for precise 3D localization of single molecules in subsequent super-resolution estimation tasks.

### 2.10. Modified deep-SMOLM CNN model architecture

We implemented a modified Deep-SMOLM (mDeep-SMOLM) neural network in Python using PyTorch for SMOLM. Model architecture and hyperparameters were specified through a JavaScript Object Notation configuration file, which defined graphics processing unit usage, random seeds, training and testing dataset directories, data loader parameters, and optimizer settings. The neural network was trained using a convolutional architecture with a stochastic gradient descent optimizer (learning rate = 0.001, momentum = 0.9, weight decay = 0.0005). A step-learning-rate scheduler reduced the learning rate by 50% every 80 epochs.

A batch size of 6 was used, a photon-intensity thresholding of 200 photons was applied to exclude detections with insufficient signal for reliable orientation estimation, and data loading was parallelized across 8 central processing unit workers. The training loop automatically split the simulated image dataset into training and testing subsets, initialized the model parameters, and optimized them with backpropagation for up to 100 epochs, with early stopping after 10 epochs if no validation improvement was observed. Model training and validation were monitored in real time using the Comet.ml [70] application programming interface, and trained models were saved in designated directories for subsequent estimation tasks.

### 2.11. 6D microscope design

**Figure 1.**
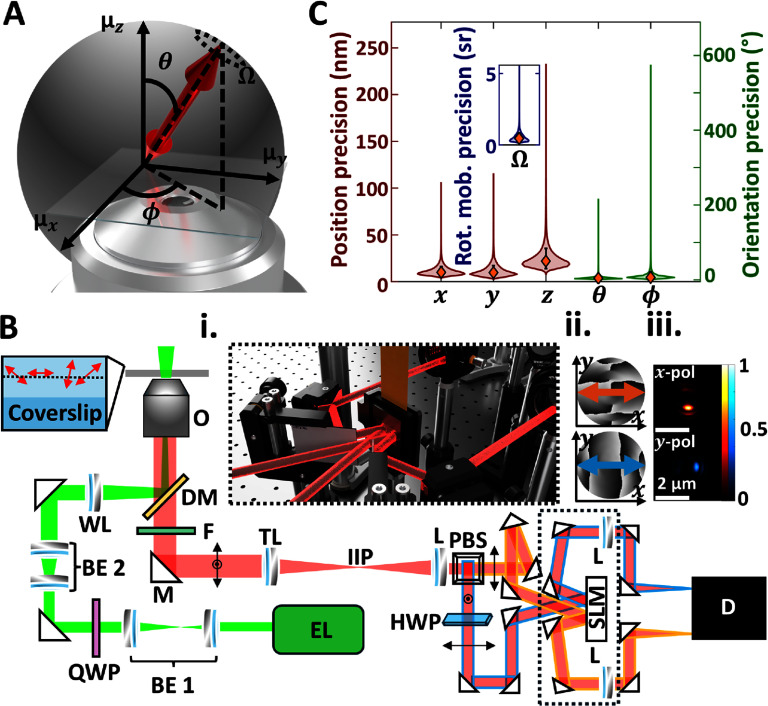
The 6D microscope constructed with an SLM, a DH phase mask, and two orthogonally polarized channels. (A) Coordinate system defining spatial ($x$, ${\text{ }}y$, $z$) and orientational ($\theta $, $\phi $, ${{\Omega }}$) parameters of a 6D measurement. (B) Schematic of the optical microscope for 6D super-resolution localization. The inset on the left shows a cartoon of the sample geometry which includes a bilayer of PMMA spincast onto a glass coverslip, with the upper layer containing RhB dyes depicted as red arrows. The black dashed line indicates intermixing between PMMA layers. EL-532 nm excitation laser, BE-beam expander, QWP-quarter wave plate, WL-wide-field lens, O-objective, DM-dichroic mirror, F-568 nm long-pass filter, M-mirror, TL-tube lens, IIP-intermediate image plane, L-lens, PBS-polarizing beam splitter, HWP-half-wave plate, SLM-spatial light modulator, D-sCMOS detector. i.) 3D view of the polarized emission incident on two different regions of the SLM at the Fourier plane. ii.) The $x$ -pol (orange) and $y$ -pol (blue) channels arrive at the SLM at the Fourier plane with the same polarization axis. iii.) DHPSFs from both polarized channels simulated in focus at ($\theta $, $\phi $, ${{\Omega }}$) = (60°, 40°, 0 sr). (C) Theoretical localization precision of all six parameters computed over n = 5.3$ \times $ 10^5^ randomly sampled emitter configurations (table S1) with 5.5$ \times $ 10^3^ signal photons and 33 background photons per pixel. Orange diamonds report median values and error bars report ±1 standard deviation. The 5th/95th percentiles are (5.6, 19) nm for $x$, (5.0, 21) nm for $y$, (14, 42) nm for *z*, (2.1°, 14°) for $\theta $, (2.4°, 25°) for $\phi $, and (0.21, 1.0) sr for Ω.

The orientation of the transition electric dipole moment of a fluorescent emitter is parametrized by its polar, azimuthal, and wobble angles, which influence its emission pattern at the detection plane. The dipole moment vector $\vec{\mu}$ of a dipole emitter, which emits photons in a toroidal pattern [23, 71], is related to its orientational components by the following expression: $\left[ {{\mu _x},{\mu _y},{\mu _z}} \right] = \left[ {\cos \left( \phi \right){\mathrm{sin}}\left( \theta \right),{\mathrm{sin}}\left( \phi \right){\mathrm{sin}}\left( \theta \right),{\mathrm{cos}}\left( \theta \right)} \right]$ [44, 51, 72]. The polar angle ($\theta $) characterizes the emitter’s deviation from the optical axis, defining its vertical orientation. The azimuthal angle ($\phi $) denotes the directional component within the ($x$, ${\text{ }}y$) plane, representing its lateral positioning (figure 1(A)). The axial position is denoted by $z$. The wobble angle (${{\Omega }}$), measured in steradians (sr), quantifies how much the emission dipole of a fluorescent molecule rotates or fluctuates during the camera integration time [73, 74]. When an emitter samples orientations within a cone of solid angle ${{\Omega }}$ during exposure, its orientational distribution can be characterized by its second moments $m = \left[ {\mu _x^2,\mu _y^2,\mu _z^2,{\mu _x}{\mu _y},{\mu _x}{\mu _z},{\mu _y}{\mu _z}} \right] \in {\mathbb{R}^6}$, related to the parameters describing dipole rotational mobility via (equations (2)**–(**4)):

\begin{equation*}\mu _k^2 = \gamma \mu _k^2 + \frac{{1 - \gamma }}{3},{ }k \in \left\{ {x,{ }y,z} \right\}\end{equation*}

\begin{equation*}{\mu _k}{\mu _l} = \gamma {\mu _k}{\mu _l},{ }k,l \in \left\{ {x,{ }y,z} \right\},{ }k \ne l\end{equation*}

\begin{equation*}\gamma = 1 - \frac{{3{{\Omega }}}}{{4\pi }} + \frac{{{{{\Omega }}^2}}}{{8{\pi ^2}}}\end{equation*}

$\langle \cdot \rangle $ represents the temporal mean over the camera integration time, and $\gamma \in \left[ {0,1} \right]$ is the rotational mobility. Fluorophores with short fluorescence lifetimes change their dipole orientation only minimally between excitation and emission, so the light they emit remains strongly polarized along the direction of absorption [75]. Varying the single dipole-emitter orientations results in perturbations of the spatial frequency distribution located at the back focal plane or conjugate back focal plane (Fourier plane) of an optical fluorescence microscope. At the image plane, this translates to variations of the dipole’s PSF shape and intensity across orthogonal polarization channels.

The 3D positions and 3D orientations of single molecules were imaged with a custom-built, inverted optical microscope (Axiovert, Carl Zeiss) in a wide-field illumination geometry (figure 1(B)). The excitation source was a 532 nm continuous wave diode laser (Compass 315 M-100SL, Coherent) directed through a quarter-wave plate and filtered by a dichroic mirror (z532/rpc633, Chroma) to induce circularly polarized light. The laser was focused on the back aperture of an oil-immersion objective (100$ \times $, 1.46 numerical aperture Apochromat, Carl Zeiss). The fluorescence from dipole emitters was captured by the objective, filtered by a bandpass filter (ZET 532/640, Chroma), then magnified and focused by the tube lens turret (*f* = 165 mm, 1.6$ \times $ magnification), forming the intermediate image plane. The fluorescence was then collected by the first lens of a 4*f* system (*f* = 200 mm). A PBS placed immediately after the first lens, separated the horizontal and vertical components of the polarized emission into $x$-polarized ($x$-pol) and $y$-polarized ($y$-pol) channels. A half-wave plate, placed with its polarizing axis at a 45° angle to the $y$-pol channel, served to rotate the vertical emission polarization into the horizontal axis. The spatial frequency space was accessed through the Fourier plane of a microscope using a 4*f*-geometry (figure 1(B i)). The development of polarization-dependent, orientation-sensitive microscopes incorporating multiple 4*f* systems has been described elsewhere [29, 42, 44]. A reflective liquid crystal spatial light modulator (SLM) (Pluto VIS 130, Holoeye), electronically programmed with two DH phase masks, was positioned at the Fourier plane of the microscope. While the SLM introduces a modest ∼5% photon loss relative to fixed dielectric phase masks [76], it offers practical advantages including electronic reconfigurability, rapid switching between PSF designs without hardware changes, and *in situ* correction of optical aberrations [77]. For photon-starved applications, a fixed phase mask may be preferable to maximize collection efficiency. The orthogonal emission paths were incident upon the SLM surface from the same direction and polarization axes, with the $x$-pol channel incorporating a DH phase mask rotated anticlockwise by 90° (figure 1(B ii)). Rotating the DH phase mask by 90° in the $x$-pol channel achieves symmetric PSF characteristics between channels. Two additional plano-convex lenses (*f* = 200 mm) focused polarized, phase-modulated emission channels onto separate regions of a back-illuminated sCMOS detector (Prime95b, Teledyne Photometrics).

## 3. Results and discussion

### 3.1. 6D microscope theoretical precision

The DHPSF model displayed high position and orientation precision. When imaging single dipole emitters, the PSF shape is modulated by the dipole’s orientation [42]. As shown in figure 1(B iii), a simulated dipole emitter at ($\theta $, $\phi $, ${{\Omega }}$) = (60°, 40°, 0 sr) exhibited distinct anisotropy signatures in the forms of lobe asymmetry and linear dichroism, which influenced theoretical precision. The theoretical precisions of our 6D estimates were quantified by the Cramér–Rao lower bound (CRLB), which sets the minimum achievable variance of an unbiased estimator for a given imaging model and noise statistics. The CRLB is obtained by inverting the Fisher information, $M{\left( {\boldsymbol{\eta }} \right)_{ij}}$. For the parameter vector $\eta = \left[ {x,y,z,\theta ,\phi ,{{\Omega }}} \right]$, the elements of the $M{\left( {\boldsymbol{\eta }} \right)_{ij}}$ were computed as (equation (5)):

\begin{align*} M{\left( {\boldsymbol{\eta }} \right)_{ij}} = \mathop \sum \limits_k \frac{1}{{{I_0}{{\left( {\boldsymbol{\eta }} \right)}_k} + b}}\left( {\frac{{\partial {I_0}{{\left( {\boldsymbol{\eta }} \right)}_k}}}{{\partial {\eta _i}}}} \right)\left( {\frac{{\partial {I_0}{{\left( {\boldsymbol{\eta }} \right)}_k}}}{{\partial {\eta _j}}}} \right)\end{align*}

where *b* is the average number of background photons in each pixel and *I_k_*(${\boldsymbol{\eta }}$) is the mean photon count in the *k*th pixel of the PSF of $I$ [78]. We report the corresponding theoretical precision as ${\sigma _{{{\boldsymbol{\eta }}_i}}} \ge \sqrt {{{[{M^{ - 1}}\left( {\boldsymbol{\eta }} \right)]}_{ii}}} $. In figure 1(C), the precision of our approach to orientation localization was calculated over 5.3$ \times $ 10^5^ 3D positions and 3D orientations with a mean of 5.5$ \times $ 10^3^ signal photons, an average of 33 background photons per pixel and an effective pixel size of 66 nm per pixel. Table S1 summarizes the parameter sampling domains and conditions. The DH mask achieved median $x$ and $y$ precision values of 10 nm and 11 nm. The DH mask also achieved a median axial precision of 22 nm (figure 1(C)). These results aligned well with literature values [58]. The median orientational precision was 4.3° and 6.4° in terms of $\theta $ and $\phi $, respectively, which indicated better precision in estimating out-of-plane orientations (figure 1(C)). The long tail in *φ* reflects low precision as *φ* approaches 0°, at which *φ* loses uniqueness [51]. The median precision of estimating ${{\Omega }}$ with these imaging parameters was 0.45, comparable to the 0.23 sr precision reported for orientation-optimized PSF designs at similar photon counts [29]. This difference reflects that the DHPSF was originally designed for 3D localization rather than orientation sensing, though it still provides sensitive rotational mobility measurements. These theoretical CRLB values provided a benchmark for achievable precision under our imaging conditions. Because the CRLB and the DL networks share the same vectorial diffraction forward model, this agreement confirms that the networks approach statistically optimal estimation performance. Independent validation of the forward model is provided by the experimental PSF comparisons, where estimated 6D parameters were used to re-simulate images that quantitatively matched experimental observations. The experimentally observed axial-to-lateral precision ratio may exceed the CRLB-predicted ratio, consistent with the well-known anisotropy of DHPSF localization precision [79] and the sensitivity of DHPSF lobe positions to small residual system aberrations such as spherical aberration, coma, and astigmatism that are not fully captured by phase mask retrieval [77]. The experimental localization and orientation errors reported later in the manuscript fall within the same order of magnitude, confirming that our fused pipeline approached the theoretical limits.

### 3.2. Fused DL pipeline and network training

**Figure 2.**
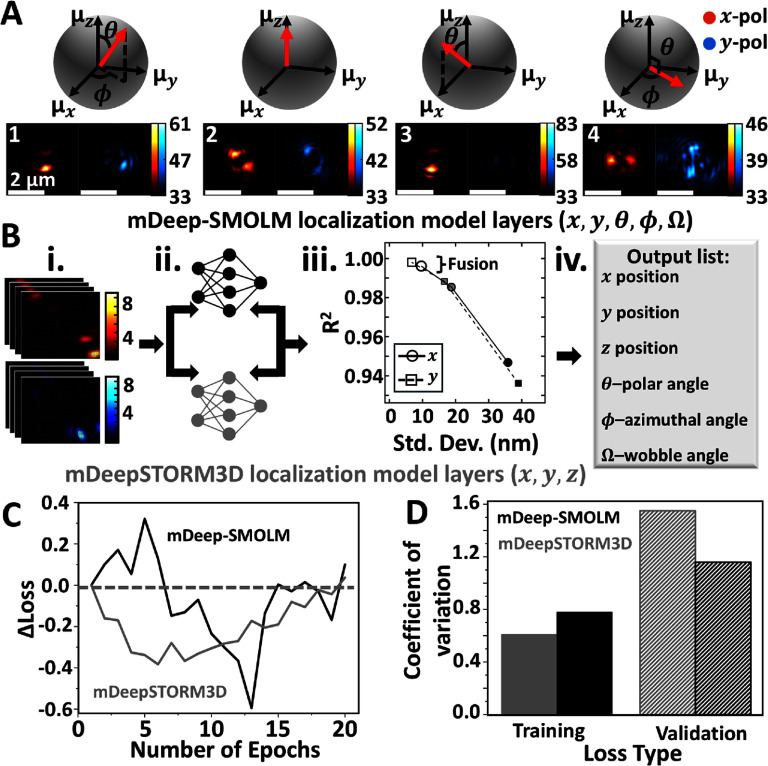
Fused processing of PSFs with DL networks enables high precision 6D parameter estimation. (A) Simulations of dipole emitters at various axial positions and orientations ($z$, $\theta $, $\phi $, $ \Omega $) generated using experimentally retrieved DH phase masks. The top row depicts the physical orientation of the dipole within the coordinate sphere. The bottom row shows simulated images at the following coordinates: 1 (0 nm, 30°, 60°, 0 sr), 2 (−400 nm, 0°, 0°, 0 sr), 3 (300 nm, 45°, 0°, 0 sr), 4 (−800 nm, 90°, 60°, 0 sr). All PSFs are shown at ($x$, $y$) = (0, 0) nm. Orientational parameters span the following coordinates: $\theta $ ∈ [0°, 90°], $\phi $ ∈ (−180°, 180°], and ${{\Omega }}$ ∈ (0, 2*π*] sr. (B) Schematic of the fused neural network pipeline for 6D dipole localization. i.) A library of $x$-pol (top, orange) and $y$-pol (bottom, blue) simulated DHPSF images was simulated with retrieved phase masks for training and validation. ii.) mDeep-SMOLM (black) and mDeepSTORM3D (gray) were applied for positional and orientational localization. iii.) Fusion of independent network predictions improves both localization accuracy (*R*^2^ correlation between ground truth and estimate) and precision (standard deviation) for lateral positions. Data points represent $x$ (circles, solid line) and $y$ (squares, dashed line) position estimates from mDeep-SMOLM (black filled), mDeepSTORM3D (gray filled), and their fusion (unfilled, enlarged). iv.) Fused results consist of the cross-validated ($x$, $y$) coordinates from both models, $z$ from mDeepSTORM3D, and ($\theta ,\phi ,{{\Omega }}$) coordinates from mDeep-SMOLM. (C) Normalized mean square error ΔLoss for mDeepSTORM3D and mDeep-SMOLM across epochs illustrates differences in training convergence. (D) CV for the two models quantified training stability and variability.

Vectorial diffraction theory enabled accurate optical simulations for 6D estimation through differences in the lobe asymmetries of the DHPSF in the orthogonal polarization detection channels (figure 2(A)). The details of the vectorial approach to optical simulations have been described elsewhere [42, 44]. Briefly, flips of the spatial frequency distributions of the vectorized $x$-pol and $y$-pol channels accounted for reflections from mirrors in the detection paths of the microscope. The PBS induced one additional reflection in the $y$-pol channel. Polarization cross-talk between detection channels was neglected in simulations, consistent with measured PBS extinction ratios exceeding 100:1. The implied cross-talk of <1% is negligible compared to the CRLB-derived precision limits (figure 1(C)). The effect of the lenses of the 4*f* system was approximated in silico via a 2D discrete Fourier transform over the phase-modulated pupils (figure S3), converting the spatial frequency information to image space. Finally, the stochastic process of photon arrival to the detector is captured by corrupting the images with Poisson shot noise [30, 80]. Consequently, different DHPSF lobe asymmetries, linear dichroism, and lobe angles were resolved at the image plane (figure 2(A)). Table S1 summarizes parameters used to compute the simulated dataset.

We fused results from mDeep-SMOLM with results from mDeepSTORM3D to achieve comprehensive 6D single-molecule localization (figure 2(B)). This fusion assigns each parameter to the network architecturally optimized for its estimation. Lateral positions are cross-validated across both networks, the axial position is drawn from the voxel-based mDeepSTORM3D, and orientational parameters are drawn from mDeep-SMOLM, which operates in the second-moment basis. Future refinements could incorporate per-estimate uncertainty weighting to further improve fusion robustness. Model training and validation were conducted with vectorial simulations (figure 2(B i)) mDeep-SMOLM inferred 5D coordinates from polarization-encoded PSFs. The network estimated the six brightness-weighted orientational second moments $m$, which enter the image formation model linearly and uniquely parameterize the mean orientation and wobble angle. mDeepSTORM3D provided nanometer-scale localization accuracy in 3D (figure 2(B ii)). Both networks independently estimated $x$ and $y$ positions from distinct optical encodings. Emitters from both networks were matched by minimizing the total lateral deviation across all possible pairings within each frame. For each frame, all possible combinations of detected molecules were evaluated, and the pairing that minimized the mean Euclidean distance in the lateral plane was selected as the optimal match. Matched pairs with lateral separation exceeding 25 nm were excluded from fusion to reject spurious pairings. Any detections not meeting this criterion were excluded from fusion. An equal-weighted fusion was chosen because quantitative analysis (figure 2(B iii) and figure S4) demonstrated that both networks yielded comparable positional variance, and that averaging reduced outliers and increased the correlation with ground truth. Relative to mDeep-SMOLM, fusion achieved *R*^2^ improvements of 5.2% ($x$) and 6.6% ($y$), with standard deviation reductions of 73% ($x$) and 83% ($y$). Relative to mDeepSTORM3D, fusion achieved *R*^2^ improvements of 1.1% ($x$) and 1.0% ($y$), with standard deviation reductions of 49% ($x$) and 60% ($y$). A weighted fusion was not employed because neither network consistently outperformed the other across frames, and their error distributions were statistically similar. Thus, equal weighting avoided introducing bias while it maximized noise robustness. Following this positional refinement, the $z$ localization from mDeepSTORM3D was fused with the orientation output of mDeep-SMOLM, yielding the complete 6D molecular parameter set (figure 2(B iv)). The total network training time was approximately 8 h on a workstation equipped with an Intel Xeon W-2255 central processing unit (3.70 GHz), an NVIDIA RTX A4500 graphics processing unit, and 64 GB of random-access memory. The training proceeded without hardware bottlenecks (figure S5).

ΔLoss, defined as the difference between validation and training loss (ideally zero, shown by the dashed line), reflected the convergence of both networks to stable behavior (figure 2(C)). This metric detects overfitting or underfitting during training. To verify that all six coupled orientation parameters converged at comparable rates, the compound loss function was additionally disaggregated into directional (*XX, YY, ZZ*) and covariant (*XY, XZ, YZ*) components (figure S6, table S2), enabling identification of anisotropic convergence behavior. Both networks initially showed large deviations from zero due to random weight initialization and high prediction error. mDeepSTORM3D exhibited a steep decline in ΔLoss in early epochs, approaching zero by epoch 10 as it rapidly learned emitter positions under sparse encoding. In contrast, mDeep-SMOLM displayed larger fluctuations because its learning objective was inherently higher-dimensional and physically more complex. mDeep-SMOLM inferred ($\theta $, $\phi $, ${{\Omega }}$) from polarization-resolved images where intensity variations were nonlinearly related to multiple orientation moments (figure S6). This coupling between angular degrees of freedom introduces a more intricate ΔLoss landscape, resulting in slower and more oscillatory convergence. After ≈15 epochs, both networks approached the baseline, with mDeep-SMOLM showing occasional excursions above zero due to mild overfitting. Training was terminated at epoch 20, where ΔLoss stabilized, which balanced generalization and prevented overfitting (figure 2(C)).

To evaluate the stability and generalization of each network during training, we quantified the coefficient of variation (CV) of the loss across epochs for both training and validation sets (figure 2(D)). The CV provides a dimensionless measure of relative fluctuation in the loss function and is defined as (equation (6):

\begin{equation*}{\mathrm{CV}} = \frac{{{\sigma _l}}}{{{\mu _l}}}\end{equation*}

here, ${\sigma _l}$ and ${\mu _l}$ denote the standard deviation and mean of the loss values, respectively, computed across all epochs for either the training or validation set. A lower CV indicates more consistent convergence, whereas a higher CV reflects greater temporal variability in the network’s learning behavior. CV values were computed from a representative training instance and repeated runs showed consistent trends. mDeep-SMOLM exhibited a higher training CV (0.78), but a lower validation CV (1.2) compared to mDeepSTORM3D (training and validation CV of 0.60 and 1.5, respectively) (figure 2(D)). This inverse CV relationship reflected the intrinsic difference in task complexity and data smoothness between the two models. mDeep-SMOLM learned a high-dimensional mapping between polarization-encoded PSFs and 5D interdependent parameters, where small weight updates affected multiple correlated outputs. As a result, mDeep-SMOLM’s training dynamics were volatile, leading to a higher training CV. However, once the network captured the underlying PSF orientation relationship, the model generalized more consistently across unseen data, yielding a lower validation CV. This behavior highlights that mDeep-SMOLM’s learning was complex during optimization but stabilized once the orientation manifold was learned. The terminal training metrics are summarized in table S2 and epoch-wise convergence behavior of the directional components of the compound loss function for mDeep-SMOLM are summarized in figure S6.

While mDeep-SMOLM achieved stable generalization after training, mDeepSTORM3D performed voxel-wise emitter localization ($x$, $y$, $z$), a simpler yet more data-sensitive task. mDeepSTORM3D’s lower training CV (figure 2(D)) indicated stable and consistent learning under sparse conditions, but the higher validation CV arose from stochastic differences in emitter density, photon counts, and $z$ distributions across validation volumes. Consequently, mDeepSTORM3D generalized less uniformly, as it was more sensitive to local variations in emitter sparsity and background noise. Training and validation loss curves for mDeep-SMOLM and mDeepSTORM3D can be found in figure S7. Overall, these CV values captured complementary learning characteristics: mDeep-SMOLM experienced complex but well-generalized learning, whereas mDeepSTORM3D demonstrated stable training yet higher validation variability due to data stochasticity. Median CV and error statistics were computed over *n* = 3.0$ \times $ 10^4^ simulated emitters for training and *n* = 3.0$ \times $ 10^3^ for validation per model to ensure statistical reliability. Quantifying CV across epochs thus provided a complementary metric for assessing the reliability and robustness of network training beyond mean ΔLoss values.

The fused pipeline performs reliably across an operating envelope spanning emitter densities from 0.10 to roughly 1 emitter/*μ*m^2^ and signal levels from approximately 5$ \times $ 10^3^–2$ \times $ 10^4^ mean signal photons per emitter at the trained background of 33 photons per pixel. Two simulation studies summarized in figure S8 establish this envelope under the same polarization-resolved DHPSF imaging model used throughout this work. The density sweep, figure S8 panels A, C, and D, shows that fused Jaccard sustains 0.71 at 0.10 emitters/*μ*m^2^ and degrades smoothly to 0.10 at 2.97 emitters/*μ*m^2^, while lateral root mean square error (RMSE) remains below 13 nm and axial RMSE below 39 nm across the same range. The photon-budget sweep, figure S8 panel B, identifies the useful signal range cited above and shows sharp degradation at backgrounds above the trained value, indicating that deployment under different imaging conditions would benefit from retraining on the target background distribution. The fused pipeline trades model dependence for flexibility. It can be adapted to arbitrary PSF designs and detection schemes through retraining, but performance is bounded by the fidelity and coverage of the training distribution, so different imaging conditions require retraining on the target conditions. Future work will incorporate benchmarking with known pupil-plane aberrations.

### 3.3. 6D localization precision

**Figure 3.**
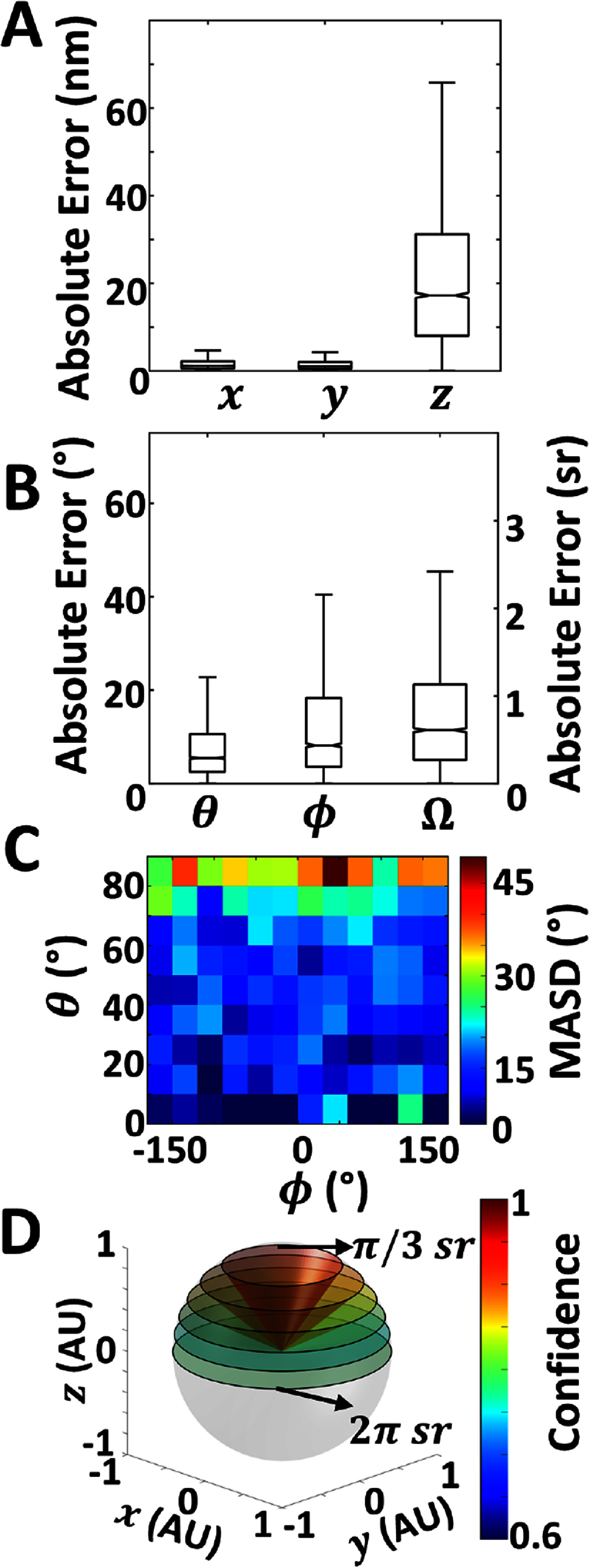
Fused 6D localization estimates single-molecule 3D spatial positions and 3D orientations with high precision. Box plots of the absolute error in (A) spatial and (B) orientational localizations. The whiskers extend to 1.5 times the interquartile range of the quartiles. (C) 2D heatmap of MASD, a combined precision measurement of $\theta $ and $\phi $. Larger regions of lower MASD values demonstrate consistently high orientational precision across many angles. (D) Hemisphere confidence map summarizing estimated ${{\Omega }}$ across the sphere. The color encodes the fraction of localizations with reliable ${{\Omega }}$ estimation in the range ${{\Omega }} \in $ (0, 2*π*] sr.

The fused mDeep-SMOLM and mDeepSTORM3D pipeline achieved high spatial precision for 6D localization. Median absolute localization errors for spatial coordinates were 1.0 nm, 1.0 nm, and 15 nm in $x$, $y$, and $z$ (figure 3(A)). Following established practice in localization microscopy, we also report RMSE values, which are comparable to CRLB-derived precision limits [81, 82]. The RMSEs for $x,y,$ and $z$ were 10 nm, 11 nm, and 33 nm. These values were consistent with the theoretical CRLB benchmarks shown in figure 1(C), where the median $x$ and $y$ precision was 10 nm, and the median $z$ precision was 22 nm. This agreement confirmed that our experimental pipeline approaches the theoretical limits under similar photon and noise conditions. The narrow interquartile ranges in figure 3(A) demonstrated consistent and reliable spatial localization across a large population of fluorescent emitters.

The fused DL workflow recovered orientation parameters with high precision in $\theta $, $\phi $, and ${{\Omega }}$. The median absolute errors for orientational coordinates were 5.3° in $\theta $, 7.0° in $\phi $, and 0.41 sr in ${{\Omega }}$, which demonstrated degree-scale recovery of molecular orientation parameters (figure 3(B)). Deviations in ${\text{ }}\phi $ were wrapped to the range [−180°, 180°] to account for the cyclic nature of this coordinate and ensure errors are measured along the shortest angular path. The RMSEs for $\theta ,{\text{ }}\phi ,$ and ${{\Omega }}$ were 13°, 45°, and 0.69 sr. The larger RMSE in $\phi $ arises from the reduced sensitivity to azimuthal orientation when dipoles are oriented nearly perpendicular to the imaging axis (high $\theta $), a phenomenon further examined in this work [42]. Regardless, these values are consistent with the theoretical CRLB benchmarks shown in figure 1(C), where the median orientational precision was 4.3° for $\theta $, 6.4° for $\phi $, and 0.45 sr for ${{\Omega }}$. This agreement confirmed that our experimental pipeline approached the theoretical limits for orientation estimation under similar photon and noise conditions. Additionally, we used the combined metric, mean angular standard deviation (MASD) [30], ${\sigma _\delta }$, to assess the combined precision in measuring $\theta $ and $\phi $ (equation (7)):

\begin{equation*}{\sigma _\delta } = 2{\mathrm{arcsin}}\left( {\sqrt {\frac{{{\mathrm{sin}}\left( \theta \right){\sigma _\theta }{\sigma _\phi }}}{\pi }} } \right) \end{equation*}

Our method produced high angular precision over a broad range of $\theta {\text{ }}$ and $\phi $ orientations, indicated by concentrated low MASD regions in the angular landscape (figure 3(C)). We observe that MASD inherently weighs $\phi $ errors by sin($\theta $), reflecting the reduced geometric significance of $\phi $ near the polar axis. The elevated MASD at high $\theta $ reflects both this geometric weighting and the increased difficulty in resolving $\phi $ for in-plane dipole orientations in our optical system. We define the confidence in ${{\Omega }}$ as (equation (8)):

\begin{equation*}{\mathrm{Confidence}}\left( {{\Omega }} \right) = 1 - \frac{{{\mathrm{mean}}\left( {\left| {{{{\Omega }}_{{\mathrm{est}}}} - {{{\Omega }}_{{\mathrm{GT}}}}} \right|} \right)}}{{{{{\Omega }}_{{\mathrm{max}}}}}}\end{equation*}

where the numerator is the mean absolute error in ${{\Omega }}$, ${{{\Omega }}_{{\mathrm{est}}}}$ is the estimated ${{\Omega }}$, ${{{\Omega }}_{{\mathrm{GT}}}}$ is the ground truth ${{\Omega }}$, and ${{{\Omega }}_{{\mathrm{max}}}}$ is the maximum possible error (2*π*) in ${{\Omega }}$. The confidence score naturally falls between 0 and 1. The uniformly high confidence values across the orientation hemisphere confirmed that this spatial precision was maintained for most dipole orientations (figure 3(D)). Correlations and deviations between predicted and ground truth values for spatial and orientational parameters from the fused results are shown in figures S9 and S10. These findings show that the fused DL workflow accurately recovered molecular orientation in $\theta $, $\phi $, and ${{\Omega }}$ with high precision.

The fused DL method achieved high recognition fidelity, with a Jaccard index of 0.74 indicating that ≈74% of emitters were correctly matched true positives from simulated images. The Jaccard index ($J$) quantitatively measured the similarity between predicted ($P$) and true ($T$) emitter sets and was computed as (equation (9)):

\begin{equation*}J = \frac{{\left| {P\mathop \cap \nolimits T} \right|}}{{\left| {P\mathop \cup \nolimits T} \right|}}\end{equation*}

where $\left| {P\mathop \cap \nolimits T} \right|$ represents the number of emitters correctly detected (true positives), and $\left| {P\mathop \cup \nolimits T} \right|$ represents the total number of unique emitters from both prediction and ground truth (true positives + false positives + false negatives). In the 2016 SMLM software challenge, the average Jaccard index across methods was **≈**0.74, with best-in-class algorithms approaching **≈**0.85, indicating that values in the 0.7–0.8 range correspond to substantial detection fidelity [60]. Therefore, **≈**0.74 denotes competitive performance in realistic localization tasks.

### 3.4. Experimental validation in PMMA films

**Figure 4.**
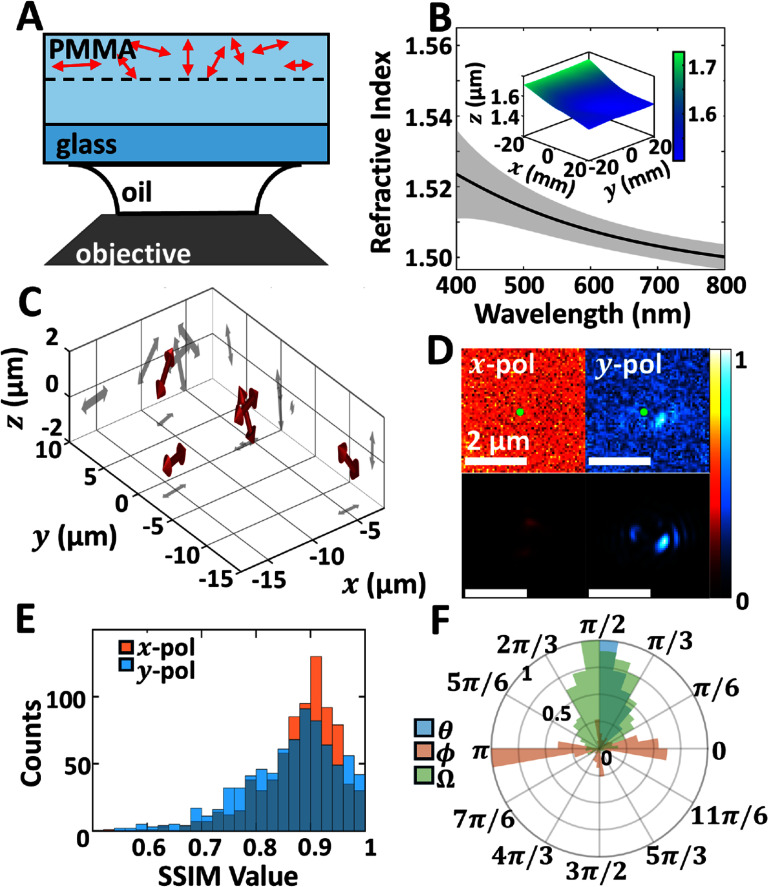
The polarization-resolving microscope and fused DL network outputs determine the 6D coordinates of single RhB molecules embedded within PMMA films. (A) Cartoon of the sample geometry, showing dipole emitters distributed within the upper PMMA film layer. Dimensions are not shown to scale. The black dashed line indicates intermixing between PMMA layers. (B) Refractive index and thickness analysis (inset) of a 20 wt% PMMA film resolved from ellipsometry characterization. (C) 3D plot of localized RhB emitters, with red dipole arrows indicating the estimated ($\theta $, $\phi $) coordinates. (D) Single-frame experimental image (top row) of a fluorescent emitter within the PMMA film. The green dots mark the estimated ($x$, $y$) coordinates of a detected molecule. The identified molecule had a $z$ coordinate of 197 nm and orientational coordinates ($\theta $, $\phi $, ${{\Omega }}$) of (58°, 94°, 0.21 sr). The bottom row shows simulated images for each channel generated from the estimated 6D parameters. (E) Histograms showing the SSIM calculated by comparing 891 crops of detected PSFs from experimental images with simulated images generated from estimated 6D parameters. (F) Polar histograms of orientation localization estimates from the emitters localized in (E).

We demonstrated 3D localization and orientation recovery of single RhB molecules in PMMA films. We imaged 165 pM RhB embedded in stacked 20 wt% PMMA (MW = 15 kD) films, where the first layer served as a control to prevent RhB interactions on the coverslip surface and the second layer contained RhB molecules. A representative widefield video showing the appearance of single RhB molecules after inclusion of the RhB‐containing PMMA layer is provided in supplementary movie 2. The sample geometry in figure 4(A) depicts a physically realistic measurement scenario in which dipoles embedded in PMMA experience a refractive index contrasting glass and oil, justifying the inclusion of layered optical effects in the forward model [44, 83–86]. When imaging the sample, fluorescent emitters were observed randomly dispersed in 3D in the film. A consistent refractive index for the 20 wt% PMMA film and a film thickness of **≈**1.6 *μ*m was resolved by ellipsometry (figure 4(B)), which validated the index and thickness parameters used in image simulation and the optical forward model (table S1). Consequently, we resolved emitter positions and orientations across the sample volume figure 4(C).

Visual and quantitative comparisons confirmed that the experimentally estimated 6D parameters and optical model jointly reproduced the observed PSF structure across orthogonally polarized channels. Figure 4(D) shows a visual comparison between manually cropped experimental PSFs (60$ \times $ 60 pixels) and simulated images generated from the corresponding 6D coordinates extracted from that experimental frame. Consistent with the comparisons shown in figure 4(D), supplementary movie 3 compiles representative PSF crops alongside simulations generated from their estimated 6D parameters, illustrating strong experiment‐model agreement. The close visual agreement between experimental PSF crops and simulated PSFs generated from estimated 6D parameters indicated that the recovered parameters produced an accurate simulated image. To further investigate the similarity between experimental images and simulated images computed from estimated 6D coordinates, we calculated the structural similarity index measure (SSIM) between thresholded experimental and simulated images using the native MATLAB function, SSIM (figure 4(E)). The SSIM is a full-reference image quality metric designed to measure the similarity between two images by comparing luminance, contrast, and structural information rather than absolute pixel errors [87]. The SSIM distributions showed that most simulated crops closely matched experimental results, with a central tendency toward high structural similarity. This quantitatively confirmed that the forward model and recovered 6D parameters reproduced experimental PSF structure across many molecules. The overlap and narrow spread of SSIM for $x$-pol and $y$-pol indicated consistent model performance across polarization channels and robustness to polarization-dependent measurement noise. As a complementary linear metric, we also computed the Pearson correlation coefficient between each experimental crop and its 6D-derived simulated counterpart (*n* = 891 detections; figure S11). The per-crop combined-channel Pearson distribution had a median of 0.25 (interquartile range [0.21, 0.31]), reflecting the single-detection shot-noise limit on pixel-wise correlation, while the aggregate Pearson on the population-average experimental versus simulated PSF reached 0.92, confirming that the forward model reproduces the population-level PSF structure independent of single-detection noise.

The 6D localizations of single RhB molecules in PMMA films reproduced literature‐reported orientation behavior. Figure S12 reports histograms of 6D localizations, whereas sharp peaks were observed for $x$ and $y$ localizations due to manually centered PSF cropping (figures S12(A) and (B)), and a broader distribution of $z$ localizations was observed because the dyes had random axial positions within the film (figure S12(C)). A total of 1412 crops were processed, from which 891 molecules were identified. Most molecules were oriented with $\theta $ approaching 90° (figures 4(F) and S12(D)). It was expected that most dyes lay approximately parallel to the glass coverslip surface for spin-coated PMMA samples [25, 88]. Additionally, while a random distribution of $\phi $ angles was expected for the RhB molecules, $\phi $ did not appear uniformly distributed around the unit circle in figures 4(F) and S12(H). Since emission appears brightest when dipoles are parallel to the optical axis [89], we expected higher estimation accuracy in $\phi $ and $\theta $ at high $\theta $ angles. We hypothesized that optical degeneracies increased estimation error in $\phi $ at high $\theta $ due to the C_2_ symmetry (180° rotation) governing the interaction between an electromagnetic field and an electric dipole [90]. Optical degeneracies occur where different emitter orientations or positions produce the same or nearly identical images [42, 91].

Simulated DHPSFs at low ($\theta \in $ [0°, 60°]), and high ($\theta \in $ (60°, 90°]) angles confirmed that optical degeneracies occurred at high $\theta $, whereas DHPSF pairs have identical lobe asymmetries and linear dichroisms at $\phi $ ≈(−180°, 0°, 180°), as well as at $\phi $ ≈(−100°, 100°) (figure S13). Consistent with this hypothesis, the correlation between simulated and estimated $\phi $ decreased from *R*^2^ = 0.57 for $\theta $ $ \le $ 60° to *R*^2^ = 0.36 for $\theta $ > 60°, confirming increased degeneracy at high ${\text{ }}\theta $ (figures S14(E) and (F)). These observations are supported by previous work involving the application of the DHPSF model to SMOLM, where it is shown that orientation estimates obtained with the DHPSF method are less reliable for single molecules with small inclination angles ($\theta $ > 75°) from the coverslip [42]. Therefore, when the estimated $\phi $ results were restricted to $\phi $ associated with $\theta \in $ (0°, 60°], the estimated $\phi $ distribution became more uniform (figure S15). Approximately 30% of emitters had $\theta $ within this range and were included in the restricted analysis. Consequently, no preferred in-plane molecular orientations were determined.

Finally, a low-angular peak in ${{\Omega }}$ (figure 4(F)) indicated tightly constrained dipoles. A rotationally fixed molecule has a cone solid angle of 0 sr, while a freely rotating molecule has ${{\Omega }}$ = 2*π* sr. Although the histograms for ${{\Omega }}$ did not peak at 0 sr, it was known that dyes in PMMA have small but non-zero rotations [43, 92, 93]. This observation was consistent with small ${{\Omega }}$ [94]. Previously reported rotationally constrained dyes in PMMA exhibit wobble cone semi-angles of $\alpha $ ≈25°–31°, corresponding to solid angles ${{\Omega }}$ ≈0.60–0.89 sr [43]. By comparison, our measured median value of ${{\Omega }}$ ≈1.5 sr indicated a larger, yet still strongly hindered, rotational mobility. The moderately larger value could arise from lower local polymer density or dye structure, or bias from manual cropping. Additionally, simulated PSFs across this wobble range (${{\Omega }}$ = 0–1.5 sr) show only subtle differences in lobe asymmetry and linear dichroism (figure S13(B)), indicating that the PSF features used for orientation estimation remain consistent across the experimentally observed range of rotational mobility. The absence of a large population of detected emitters with ${{\Omega }}$ ≈0 reflects experimental limitations such as finite signal-to-noise ratio, background fluorescence, and residual PSF distortions, which bias orientation estimates toward small nonzero wobble values even for nearly fixed dipoles, rather than indicating a failure of the model or analysis.

To test the pipeline on a distinct, curved 3D geometry, we imaged single Nile red molecules in a DPPC membrane coating a 2 *µ*m silica bead (figure S16). Pooling fused 6D detections across sequential movies of Nile red binding transiently to one bead and fitting the localizations to a sphere returned a radius of 1155 nm, in agreement with the nominal bead radius and confirming that the recovered positions decorate the membrane surface. Despite operating outside the training distribution in both photon budget and sample medium, the recovered orientations still show a clear angular dependence across the bead surface. A representative movie of single Nile red molecules blinking on the DPPC-coated beads is provided in supplementary movie 4. The recovered wobble was broad (median Ω = 2.88 sr). Because every detection in this dataset fell roughly an order of magnitude below the trained signal level, *γ* is expected to be biased low (see figure S16 discussion) and we interpret the measured Ω as an upper bound rather than a calibrated value, consistent with the more constrained dipole expected for Nile red in a DPPC membrane [22]. This out-of-distribution behavior reinforces that deployment under different photon budgets benefits from retraining on the target conditions.

Extending this static 6D localization approach to time-resolved tracking is an important future direction that introduces additional considerations. Shorter camera integration times will reduce the photon budget per frame, degrading both spatial and orientational CRLB precision. The large footprint of the DHPSF further compounds this by distributing photons across many pixels, reducing per-pixel signal-to-noise. Brighter and more photostable fluorescent probes [95] would offer a direct route to maintaining orientation sensitivity at shorter exposures, while wide-field high-speed acquisition strategies such as SpeedyTrack [96] could further improve temporal resolution with EM-CCD detection. Furthermore, the electronic reconfigurability of the SLM will enable rapid switching between PSF designs optimized for different tradeoffs between temporal resolution, spatial precision, and orientational sensitivity without hardware modifications.

## 4. Conclusions

This work establishes a robust DL framework for comprehensive 6D single-molecule localization by fusing mDeep-SMOLM and mDeepSTORM3D networks to simultaneously recover 3D spatial positions and 3D orientations of fluorescent emitters. Through polarized wide-field microscopy with DHPSFs, vectorial diffraction theory-based simulations, and fused DL outputs, we achieved high precision in both localization and orientation estimation. CRLB analysis showed that the DH mask achieves median spatial precisions of 10 nm ($x$, ${\text{ }}y$), 22 nm ($z$), and orientational precisions of 4.3° ($\theta $), 6.4° ($\phi $), and 0.45 sr (${{\Omega }}$) under 5.5$ \times $ 10^3^ signal photons and 33 background photons per pixel. Estimations were validated by stable network convergence and a low CV across training epochs (mDeep-SMOLM: CV = 0.78 training, 1.2 validation; mDeepSTORM3D: CV = 0.60 training, 1.5 validation). Our method demonstrated strong detection fidelity with a Jaccard index of 0.74 and successfully reproduced literature-reported orientation behavior when applied to single RhB molecules in PMMA films. Quantitative comparisons between experimentally measured 6D parameters and theoretical optical models confirmed accurate recovery of molecular dipole orientations through polarization-resolved PSF asymmetries.

Although the fused 6D pipeline provides a practical and experimentally accessible route to simultaneous 3D spatial and 3D orientational localization, it does not achieve the highest possible orientational precision. Alternative phase-engineering approaches optimized specifically for orientation sensitivity have demonstrated superior angular precision: the unpolarized vortex PSF achieves **≈**3°–6° orientation precision for rotationally constrained emitters with 4$ \times $ 10^3^ signal photons [43], the DL-optimized Arrowhead PSF achieves **≈**5° orientation precision over a 1 *μ*m depth range [51], the raMVR microscope attains 2.0° orientation over a 1.5 *μ*m depth range [29], the pixOL microscope achieves 4.1° orientation and 23.2 nm lateral localization precision with 2.5$ \times $ 10^3^ detected photons over a 700 nm depth range [97], and the Tri-spot PSF corrects orientation-induced localization bias from 30 nm to 7 nm [93]. Notably, the unpolarized vortex PSF produces a compact footprint only 1.5–2$ \times $ larger than a standard PSF [43]. In contrast, our DH-based approach and other engineered orientation-sensitive PSFs such as the bisected pupil [98] and Tri-spot [93] produce larger PSF footprints than the vortex PSF (4–16$ \times $ the Rayleigh criterion) [43]. This increased footprint reduces signal-to-background ratio and limits emitter density per frame [43]. Furthermore, previous work demonstrated that the DHPSF encodes orientation information via lobe asymmetry and polarization-dependent intensity differences [42]. However, the orientation estimation in this work relied on heuristic indicator of linear dichroism and lobe asymmetry, extracted by fitting images to a double-Gaussian model that does not capture the finer diffraction features of the true vectorial PSF. Additionally, orientation was retrieved through look-up table interpolation with empirical aberration handling, rather than a systematic forward-modeling framework with explicit CRLB-based precision analysis for orientation parameters [42]. In contrast, our approach employs a fully quantitative framework: vectorial diffraction simulations, CRLB-derived precision bounds, and deep-learning-based estimation that jointly recovers all six parameters. This distinction underscores that, while DH-based 6D imaging does not represent the theoretical limit of orientational precision, our fused optical-computational pipeline provides a balanced, analytically grounded solution for high-precision 6D localization in practical experimental settings.

Our method resolves heterogeneity that is separable into independent detection events in space or in time but cannot resolve heterogeneity that co-occurs or interconverts within a single detection. The forward model used to generate the training set treats the emitter as a single fluorescent dipole emitting within one spectral channel, with rotational mobility described by a uniform conical aperture [73], so the network associates each input PSF with one set of position and orientation parameters drawn from this single-state space. Mixtures of spectrally distinct probes are also not separated. Chromatic differences in the phase mask, channel splitter, and dichroic responses produce subtly different PSFs [37, 38] that the current single-spectrum model cannot decompose. Sub-exposure interconversion between orientational states is likewise unresolvable, producing an apparent intermediate orientation with inflated wobble, indistinguishable from a single, more mobile dipole [74]. By contrast, a probe occupying two or more stable orientational states that are spatially or temporally separable, such as bimodally bound dye in distinct binding pockets, produces independent detections at each state. The modes appear in the aggregated population, as demonstrated for single-molecule dipole orientation imaging of probe–substrate interactions on 2D crystals [17]. For single-molecule polarized imaging of amyloid fibrils, where two distinct binding configurations of the same fluorescent rotor probe an in-plane mode at $\theta \approx $ 90$^\circ $ and an out-of-plane population at $\theta $ $ &lt; $ 60° constituting roughly 60% of detected events were quantitatively resolved as separable sub-populations in the aggregated orientation distribution [99]. Characterization of within-detection mixtures would therefore require training data spanning the joint probe-orientation-spectrum distribution of interest, an approach explored recently in parameter-aware deep-learning frameworks for SMLM [57].

Within the model’s coverage, per-detection credibility is established by three filtering layers. A 200-photon intensity threshold excludes detections with insufficient signal for reliable 6D estimation, a 25 nm lateral cross-validation gate in the fusion step requires that mDeepSTORM3D and mDeep-SMOLM agree before a detection is reported, and the operating-envelope characterization in figure S8 defines the signal-photon and emitter-density regime in which the population-level statistics are reliable. Together these filters help distinguish credible rare events from spurious detections, but they do not yet provide an explicit per-detection confidence score. A calibrated per-detection uncertainty estimate is a target for future development and would be the appropriate framework for formally evaluating individual detections, including rare ones, against a stated reliability threshold.

Hence, this fused pipeline is a powerful tool for probing nanoscale molecular organization and dynamics in complex systems. Future work will extend the current static 6D localization to dynamic tracking across frames, enabling real‐time observation of rotational and translational diffusion in heterogeneous environments. Dynamic tracking will transform the current approach from a proof‐of‐concept into a versatile platform for studying biological systems, as well as separations [100] and energy storage materials [101], where orientation-resolved measurements can reveal transport mechanisms and structural heterogeneity at the single-molecule level.

## Data Availability

The data supporting the findings of this study are available in the Illinois Data Bank at [DOI: https://doi.org/10.13012/B2IDB-6080564_V1], under a CC BY license. The analysis pipeline, training and evaluation code, and trained model weights for mDeep-SMOLM and mDeepSTORM3D are openly available on GitHub at https://github.com/LandesLinkLab/6D-microscopy, with large mDeepSTORM3D data files (Din.mat, Dtar.mat, target_bol.mat, pred_bol.mat) attached to the v1.0 release at https://github.com/LandesLinkLab/6D-microscopy/releases/tag/v1.0. Supplementary data 1 available at: https://doi.org/10.1088/2050-6120/ae8eff/data1. Supplementary data 2 available at: https://doi.org/10.1088/2050-6120/ae8eff/data2. Supplementary data 3 available at: https://doi.org/10.1088/2050-6120/ae8eff/data3. Supplementary data 4 available at: https://doi.org/10.1088/2050-6120/ae8eff/data4. Supplementary data 5 available at: https://doi.org/10.1088/2050-6120/ae8eff/data5.
